# The power of stories in Pediatrics and Genetics

**DOI:** 10.1186/s13052-016-0241-z

**Published:** 2016-04-05

**Authors:** John M. Opitz, Lorenzo Pavone, Giovanni Corsello

**Affiliations:** Pediatrics (Medical Genetics), Pediatric Pathology, Human Genetics, Obstetrics and Gynecology, School of Medicine, University of Utah, Salt Lake City, UT USA; Unit of Pediatrics and Pediatric Emergency, University Hospital “Policlinico-Vittorio Emanuele”, Università di Catania, Catania, Italy; Department of Sciences for Health Promotion and Mother and Child Care, Università di Palermo, Via Alfonso Giordano, 3, 90127 Palermo, Italy

## Abstract

On the occasion of the opening ceremony of the 43rd Sicilian Congress of Pediatrics, linked with Italian Society of Pediatrics SIP, SIN, SIMEUP, SIAIP and SINP, held in Catania in November 2015, the Organizing Committee dedicated a tribute to Professor John Opitz and invited him to give a Masters Lecture for the attendees at the Congress. The theme expounded was “Storytelling in Pediatrics and Genetics: Lessons from Aesop and from Mendel”. The contribution of John Opitz to the understanding of pediatric clinical disorders and genetic anomalies has been extremely relevant. The interests of Professor John Opitz are linked not only to genetic disorders but also extend to historical medicine, history of the literature and to human evolution. Due to his exceptional talent, combined with his specific interest and basal knowledge in the genetic and pediatric fields, he is widely credited to be one of the best pediatricians in the world.

## Background

**Fig. 1:**
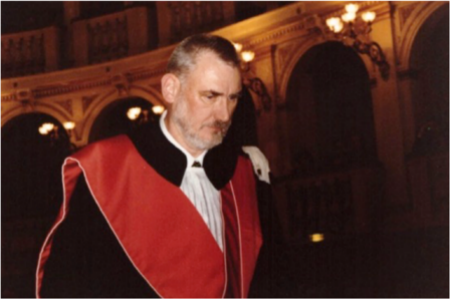
Prof. John M. Opitz

On the occasion of the opening ceremony of the 43^rd^ Sicilian Congress of Pediatrics, linked with SIP, SIN, SIMEUP, SIAIP and SINP, held in Catania in November 2015, the Organizing Committee dedicated a tribute to Professor John Opitz (Fig. 1) and invited him to give a Masters Lecture for the attendees at the Congress. The connection with Professor Opitz was carried out directly through Skype and the theme expounded was “Storytelling in Pediatrics and Genetics: Lessons from Aesop and from Mendel”. For most of the audience the chance to see his image and hear his voice was a tremendously emotional experience.

John Opitz was born in Hamburg in 1935, but at a young age he moved with his mother from Germany to the US. Soon after his arrival in the US he attended the University of Iowa, where he was introduced to the famous embryologist, geneticist, and zoologist professor Emil Witschil, with whom he started to work scientifically. In Iowa, one of the most ancient and accredited Universities in the US, he completed the medical course where he was appreciated for his scientific capacity by all the teachers of this University including the pediatrician Professor Hans Zellweger, who was also in Iowa having been formerly at the famous school of Fanconi in Zurich. Soon after, he moved from Iowa City to Madison where Klaus Patau was teaching and subsequently studied in Wisconsin with David W Smith. During his long stay in Wisconsin he continued to develop his great interest in fetal/pediatric genetic pathology and explored the concept of atavism and the hypothesis of human developmental fields in congenital disorders. Accepting the invitation of Dr. Philip Pallister he moved from Wisconsin to the appointment as Director of the Shodair Montana Regional Genetic Service Program in Helena, Montana, where he remained for 8 years. Subsequently, he joined the University of Utah School of Medicine as Professor of Pediatrics in the Division of Medical Genetics.

The contribution of John Opitz to the understanding of pediatric clinical disorders and genetic anomalies has been extremely relevant. The interests of Professor John Opitz are linked not only to genetic disorders but also extend to historical medicine, history of the literature and to human evolution. Due to his exceptional talent, combined with his specific interest and basal knowledge in the genetic and pediatric fields, he is widely credited to be one of the best pediatricians in the world. He has received numerous awards and distinctions, and honorary degrees from the University of Montana, Kiel, Copenhagen and Ohio, to mention just a few. He was designated winner of the William Allan Award of the American Society of Human Genetics and in 1999, at the instigation of Professor Liborio Giuffrè from Palermo, he was awarded with the ‘Laurea Honoris Causae’ at Bologna University.

Sicilian Universities have kept in contact with this great scientist in the course of the years. With the collaboration of the Professor Giovanni Neri from the Catholic University of Rome, the three Sicilian universities and the Oasi of Troina regularly invited him in turn each year, with his prompt approval. Some of us had the opportunity to assist with the physical examinations performed by Professor Opitz during his stay in Sicily. He showed his great talent with scientific and clinical competence, professionalism, empathy and delicacy in talking with the parents of the affected children.

We were fortunate indeed to have the opportunity to take part in his clinical performance and each time it was a lesson in human behavioral and clinical professionalism.

Prof. Giovanni Corsello and Prof. Lorenzo Pavone

Motto:

“We believe in the power of Science, Exploration, and Storytelling to Change the World.”

Mission Statement, National Geographic Society of the USA 288(4), Oct 2015 [16].

### Introduction

“… to change the world,” we all hope, (contrary to ISIS), for the better.

First, allow me to express to my most distinguished colleagues and friends of decades Giovanni Corsello of Palermo and Lorenzo Pavone of Catania, and the members of the Organizing Committee, my gratitude for their most gracious invitation to participate at this congress of the Italian (Sicilian) Society of Pediatrics. Inability to participate personally for various reasons has been a strong impetus for me to prepare this review. To pay homage also to the Italians of immortal fame who were storytellers, e.g. Boccaccio, Petrarch, Dante, Tasso and those working more recently as scientists e.g. Italo Barrai, Angelo Serra, Paul Polani, Luigi Cavalli-Sforza, Mario Capecchi, Guido Pontecorvo, Salvador Luria, Renato Dulbecco, a.o., who had made lasting contributions to genetics, basic, human and medical.

**Fig. 2:**
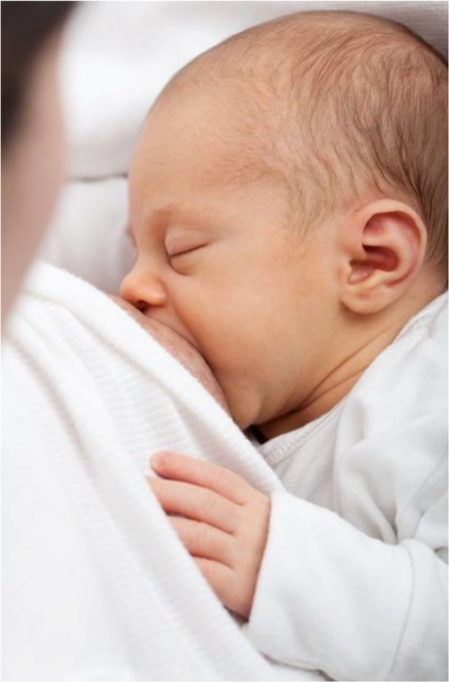
Nursing infant. CC0 Public Domain

Now, addressing mostly pediatricians, allow me to begin with storytelling. The difference between pre- and postnatal conditions is that a formerly muffled voice is now clear, and unless the infant is deaf it will be exposed immediately after birth to the emotional content of mother’s vocalizations. This, and the need and ability of baby to nurse (Fig. 2), creates the closeness necessary for the normal psychomotor development of the infant. And that includes speech development.

**Fig. 3:**
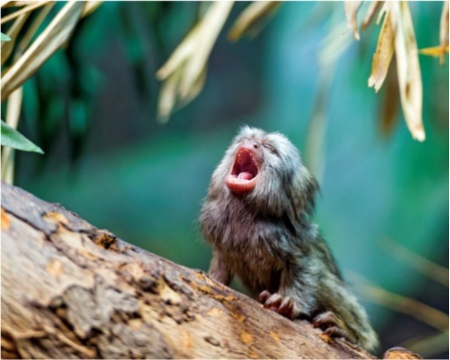
“Baby marmoset with open mouth” by Tambako The Jaguar is licensed under CC BY-ND 2.0

**Fig. 4:**
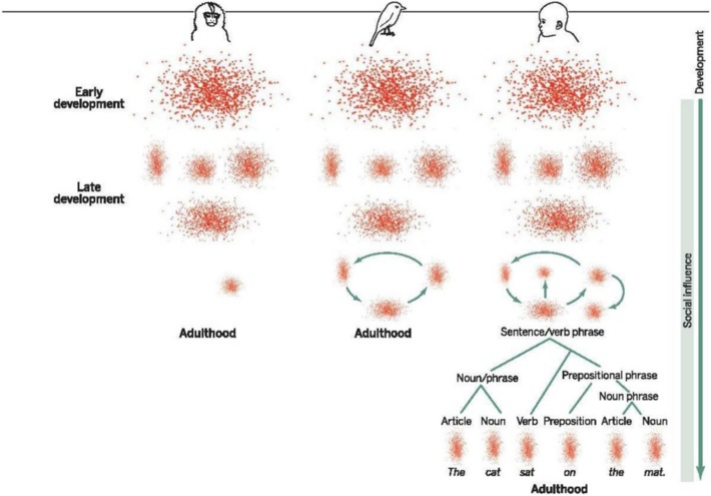
Figure/Image ‘Similar yet different,’ from Science, “Marmoset kids actually listen”, Daniel Margoliash and Ofer Tchernichovski, Vol. 349 no. 6249 pp. 689. Reprinted with permission from AAAS

In a convincing recent study from Princeton University, Takahashi et al. [22] studied voice development in marmosets (*Callithrix jacchus*) beginning with cries of distress to cooing, then babbling as the voice apparatus matures. An important component of that process is mother’s vocal feedback apparently modifying the infant’s voice production. To me, incredibly, it was thought, until recently, that nonhuman primates underwent “few or no production-related acoustic changes during development” and that “any such changes [were] thought to be impervious to… feedback.” Takahashi et al. [22] used a quantitative tracking and recording approach and biomechanical modeling and showed something that could have been done 50 years ago, namely that vocalizations in infant marmosets (Fig. 3) undergo “dramatic changes” with age not solely attributable to “simple consequences of growth.” Also, Takahashi et al. [22] found that [mother’s] feedback influences the rate of vocal development making the marmoset a very useful model of human speech development. Normal integration of a growing child into a family toward eventual kindergarten and school entry requires normal speech development. In the editorial comment on the Takahashi et al. report, Margoliash and Tschernichowski [13] address its evolutionary implications. They note that the early stages of speech development are “remarkably similar in monkey, bird and humans” and that at the beginning vocal sounds are “highly variable and unstable” constituting, or clustering into, a single diffuse, nonspecific cloud of cries (Fig. 4). As the organism begins to mature this cloud begins to separate into several distinct and separate vocalizations, indicating a transition from a continuous graded signal “to a weakly symbolic vocal performance” which undergoes further differentiation and selective attrition.”

A process for combinatorial capacity emerges, “prolonged and extensive in humans leading eventually to spoken language.” What I find remarkable from an evolutionary perspective is that the “brainstem – midbrain systems for call production are common in vertebrates.” Margoliash and Tschernichowski [13] postulate that species- specific differences in vocal production “might have evolved through gradual increase in the interaction between these primitive brain structures and the…. forebrain” with resulting species – specific differences in combinatorial and symbolic processes. Thus, the results of Takahashi et al. seem to identify an ancient capacity for vocal learning refined by an enlarging human brain continuing an evolutionary process that led to communication in other animals.

Since, in the mouse, adult cortical plasticity depends on stimulation during an early postnatal critical period [9], it seems prudent to assume the same for humans and to promote it within reasonable physiological limits.

**Fig. 5:**
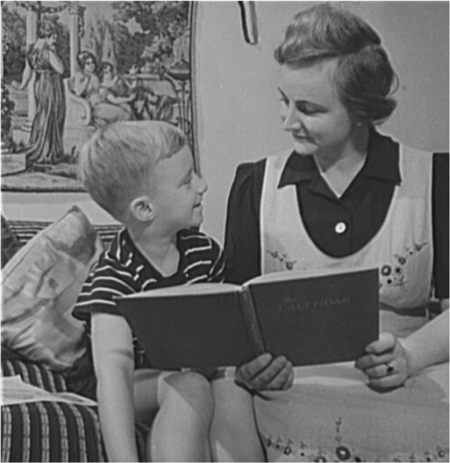
[New York, New York. Children’s Colony, a school for refugee children administered by a Viennese, German refugee mother reading to her son] Library of Congress, Prints & Photographs Division, FSA/OWI Collection, [LC-USW3-009950-E]

**Fig. 6:**
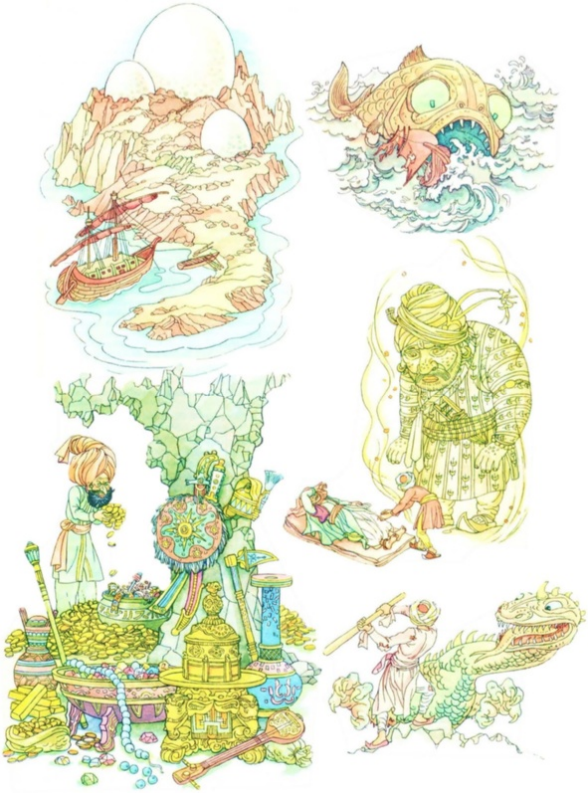
Composite of images by Martin and Ruth Koser-Michaëls illustrating [10] Erzählungen aus Tausend und eine Nacht. Munich, Droemersche Verlagsanstalt Th, Knaur Nachf

Thus, what began as that crucially necessary, life-promoting process of mother nursing and singing to her infant, gradually becomes an ever more sophisticated verbal interaction with the infant sitting on her lap after nursing while mother reads to baby *undisturbed by telephone and “texting.”* With increasing age and maturity these stories become fairy tales, finally fables with a “moral” (Fig. 5) at naptime or nighttime mother may lie down with her child until it is asleep *telling* stories instead of reading them with resulting variations on the theme, no two alike in verbal tone and structure. As the child begins to crawl and to pull books from the bottom shelf to peruse by itself, it may be, as I was at an early age, enchanted by the images in the book (Fig. 6) helping it to connect ever more firmly image with symbol and its meaning, remembering mother’s telling and retelling of the story. Thus, take fable 117 attributed to Aesop, about the hare and the tortoise [1] with illustrations by Arthur Rackham, perhaps with image of the bunny snoring in the middle of the onion patch as turtle plods by to the goal. A really good fable has staying power, retold over centuries in different languages and in different styles. Thus, fable 117 turned up in Low German in my father’s primer retold now via the brothers Grimm as the race between the hare and the hedgehog, moral now not “slow and steady wins the race” but: Since that time no rabbit in the heath of Buxtehude has ever again challenged a hedgehog to a race [4]. No parent I know or have known, or pediatrician for that matter, can deny the extraordinary value and power of reading or storytelling in raising a child. And for that boy or girl to finally discern, to personal benefit, the moral of the story, or the “story behind the story”.

## Darwin

**Fig. 7:**
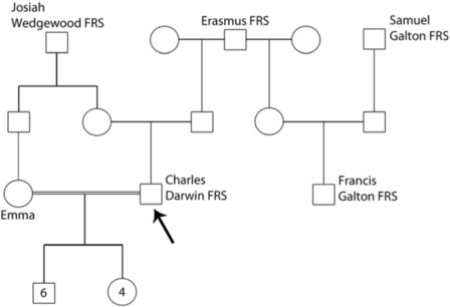
Relationship (in outline) of Darwin and Galton, drawn from data in [6]

Darwin (1809–1882) and Mendel (1822–1884) were contemporaries, the latter having read and annotated the 3^rd^ (English, 2^nd^ German) edition of the *Origin of Species…* (1859) while Darwin and Galton (Fig. 7) were and remained ignorant of Mendel’s work [7]. Mendel left no comment on Darwin, a theologically prudent thing to do at the time, given Mendel had already caused enough trouble for the Empire and Church by his stubborn refusal to pay the state-imposed tax on church property, specifically monasteries. But as Darlington [6] has pointed out, Darwin’s potential response to Mendel can be guessed from his response to Hooker (on September 13, 1864) when the latter told him of the work of Charles Naudin (1815–1899) who had already, in 1852, postulated evolution by natural selection, and published (Naudin, 1864) results on plant hybridization, anticipating in essence those of Mendel [17]. Darwin:

“I cannot think it will hold. The tendency of hybrids to revert to either parent is part of a wider law… that crossing races as well as species tends to bring back characters which existed in progenitors hundreds and thousands of generations ago. Why this should be so, God knows” [6].

**Fig. 8:**
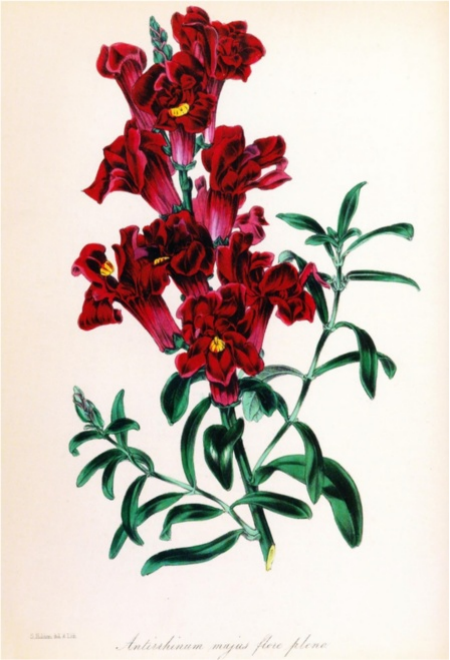
Snapdragon, *Antirrhinum majus*, here with zygomorphic blossom structure crossed with peloric variant, by Darwin [8] with all progeny in a zygomorphic form, which when selfed, yielded offspring in a ~3:1 ratio of zygomorphic (n:88) to peloric (n:37) blossom structure, so close, without recognizing it, to Mendel’s results. From Thomé OW, Migula M. 1886–1934. Prof. Dr. Thomé’s Flora von Deutschland, Österreich und der Schweiz, in Wort und Bild, für Schule und Haus. Retrieved from: http://biodiversitylibrary.org/bibliography/5360#/summary

**Fig. 9:**
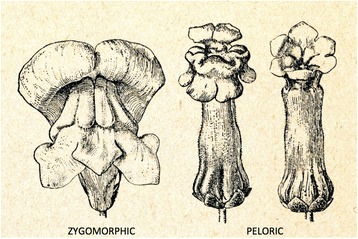
Zygomorphic (left image) and peloric forms (two right images) of the snapdragon crossed by Darwin and cited by him in 1868. The peloric form was fertile and hereditary. The peloric deviation from the normal zygomorphic form in flower morphology was, according to Stubbe [21], discovered in 1742 and described by Linné (1744) in *Linaría* (toadflaxes and snapdragons), and was one of the first observations in the plant Kingdom raising doubt about the constancy of species (or as Línné had once put it: “*Species tot numeramus, quot diversae formae in primítione sunt creatae”* (there are as many species as forms first created)

Darwin, preoccupied as he was with heredity and inheritance, mentions in *The Variation of Animals and Plants under Domestication* [1868, Vol II, p 70, i.e. 2 years after Mendel] that he had crossed zygomorphic and peloric forms of *Antirrhinum*, the snapdragon (Figs. 8 and 9). He found that *all* the offspring of this cross were zygomorphic in blossom structure. Self-fertilization of these zygomorphic hybrids yielded 88 zygomorphic and 37 peloric plants, close to a 3:1 ratio, not analyzed or interpreted further by Darwin [21], p 145. Darwin also crossed varieties of peas.

Thus, in *“The Variation of Plants and Animals under Domestication”* (including The Provisional Hypothesis of Pangenesis) of 1868 Darwin ended up postulating (again, after Hippokrates ~ 400 BCE) the existence of “gemmules” produced by every part of the body affecting germ cells and offspring modified by the parental internal and external conditions of life. As Hippokrates put it: “… the seed comes from all parts of the body, healthy seed from healthy parts, diseased seed from diseased parts” quoted in [6]. In other words, pure and plain Lamarckism i.e. Darwinism imposed on a theory of blending inheritance.

## Mendel

For me, this is the final time this year to recollect 2015 as the sesquicentenary of Mendel’s two presentations, on 2/8 and 3/8/1865 of his *Versuche über Pflanzenhybriden* published in 1866 as the methodologically soundest cornerstone of the science of genetics.

**Fig. 10:**
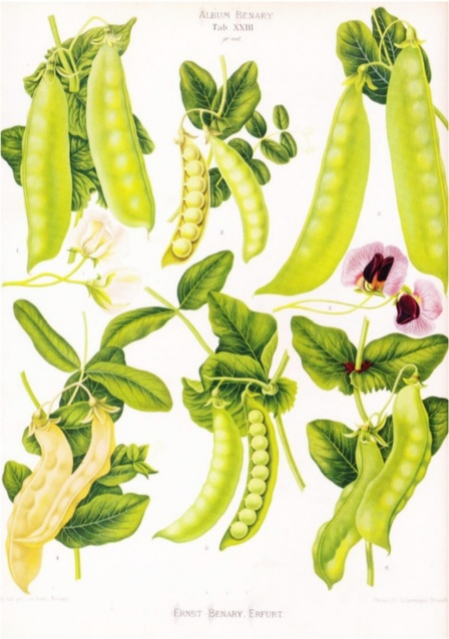
Illustration of peas from the Benary seed catalogue of 1876. From Thomé OW, Migula M. 1886–1934. Prof. Dr. Thomé’s Flora von Deutschland, Österreich und der Schweiz, in Wort und Bild, für Schule und Haus. Retrieved from: http://biodiversitylibrary.org/bibliography/5360#/summary

**Fig. 11:**
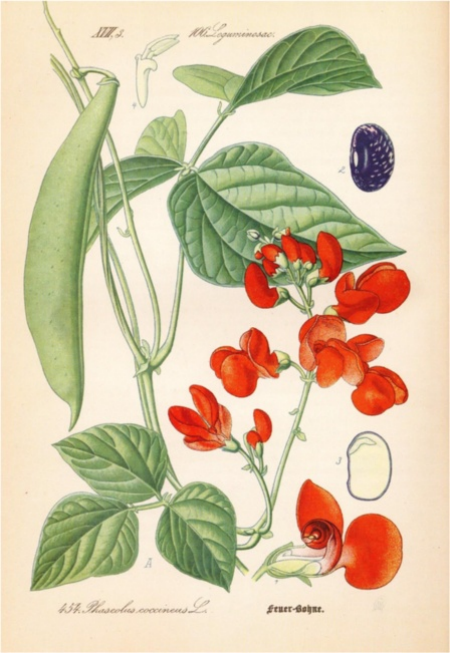
Mendel confirmed the results on peas in the bean, here *Phaseolus coccineus L*, also with leguminous blossom structure

**Fig. 12:**
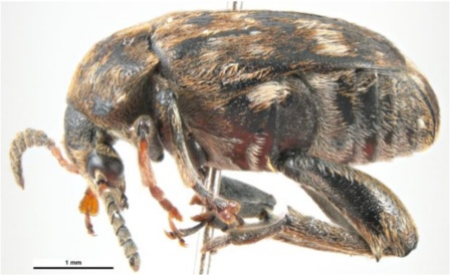
*Bruchus pisi,* or *B. pisorum*, pest of pea plants, subject of Mendel’s report of 1854 while at the University of Vienna. Image # 5311075, Pest and Disease Image Library, Bugwood.org

Mendel’s research may very well have been inspired by boyhood experiences and observations cultivating garden peas, then and now an important food crop in Moravia, and/or by his mother, a gardener’s daughter, who was known for her beautiful gardens of ornamental flowers. During his studies of theology and philosophy (1844–1845) in Brünn (Brno) Mendel also completed a one-year training course in agronomy and a one semester seminar in horticulture of grapes and fruit trees [12, 23]. In any event, something in his early years must have tipped Mendel off as to the suitability of the garden pea (Fig. 10) or bean (Fig. 11) as experimental subjects, and certain prior observations must have urged Mendel into a cross-hybridization approach to test hypotheses in his mind on the inheritance of specific traits in peas. The fact that while attending the University of Vienna (1851–1853) Mendel published (1854) a note on the pest *Bruchus pisi*, the pea borer which periodically devastated the crop (Fig. 12), testifies to his early preoccupation with the garden pea [15]. Even more important was Mendel’s education in mathematics at the university, specifically the field of combinatorics.

My late and most distinguished friend and colleague at the University of Wisconsin, Charles W. Cotterman, expert at combinatorics, left me xerox copies of 2 of the textbooks Mendel used during his studies (primarily of physics) at the University of Vienna.

- Öttinger L. 1837. *Die Lehre von den Combinationen nach einem neuen Systeme bearbeitet und erweitert*. Freiburg, Gebrüd. Groos. Öttinger was then Professor of Mathematics at the University of Freiburg.
  and:
- Ettinghausen A von. 1826. *Die combinatorische Analysis als Vorbereitungslehre zum Studium der theoretischen höhern Mathematik*. Wien, JB Wallingshauser, with a note by Cotterman that this copy of the work had been inscribed by the author to a “dear friend Stephan, April 7, 1863.”

Both of these were highly advanced treatises. Von Ettinghausen was one of Mendel’s teachers at the University of Vienna, successor of Doppler as Professor of Physics.

In any event, it seems that as early as 1851 or 1852, Mendel had a research plan in mind. He returned to the Augustinian monastery of St. Thomas of Alt-Brünn on 21 Juli 1852; soon thereafter (Mawer, 2006) Mendel ordered his seed peas a.o from the Benary company in Erfurt, Germany, the same firm from which both of my grandfathers ordered their seed a lifetime later.

**Fig. 13:**
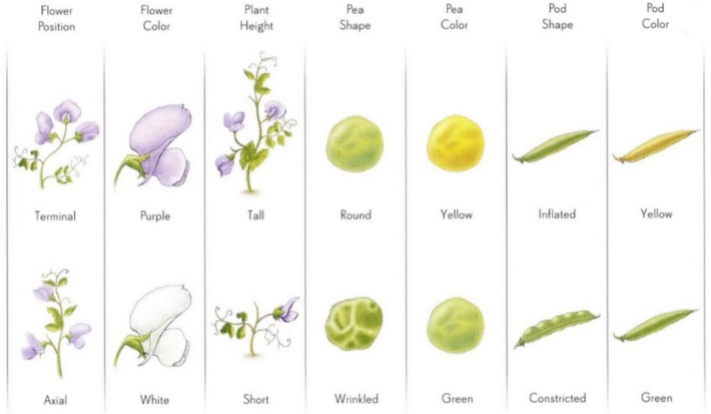
The 7 traits in peas on which Mendel eventually settled for his studies. From [14]

Mendel’s discoveries (or confirmations of his hypotheses) was one of the most striking examples ever in biology of a treasure trove of experimental data “seeking” and finding a phenomenally prepared mind. Mendel had ordered 34 kinds of pea seeds, 33 turned out to be reliably transmitting types with constant characteristics. He then selected 22 sorts which he tested during the two preliminary years 1854–1855, eliminating all but 7 traits that differed distinctively from each other as complementary trait pairs, and from the other 6 traits, namely (Fig. 13):

- Aa^1^: seed shape, round vs wrinkly
- Bb: Seed color, yellow vs green
- Cc: Pod color, gray brown vs white with blossom color violet red vs white
- Dd: Pod form, smooth vs serially constricted
- Ee: Color of immature pod, green vs yellow
- Ff: Position of flowers, axial vs terminal
- Gg: Length of plant axis, tall vs short

**Fig. 14:**
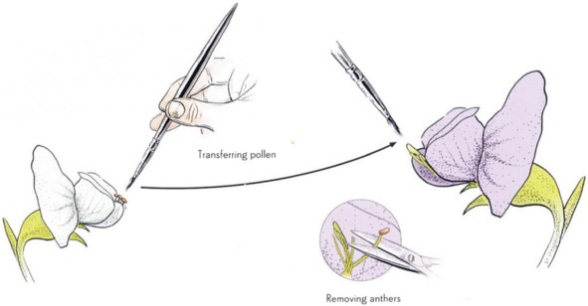
Cross-pollination in peas. From Mawer 2006

Mendel also appreciated, as a great advantage of this species, the ease with which it can be cross fertilized (Fig. 14).

Clearly, Mendel knew what he was doing. Also, he was fortunate in that all traits were unlinked except for 2 (Gg, Dd) on the same chromosome (4), but far enough apart so as to appear unlinked in his work [23].

He finished in 1864, analyzed the data, and prepared his manuscript in his characteristically beautiful handwriting. One can imagine that writing the paper for presentation in two sessions on 2/8 and 3/8/1865 and for publication the following year in the proceedings of the Natural History Society of Brünn must have given Mendel considerable headaches. Because for both purposes he had to condense, hence select, hence leave off all of those data, we now wish to know and now lost as Mendel’s successor as Abbot destroyed (burned) what was left at Mendel’s death. It must be remembered that his two presentations were a *didactic* exercise, structured to convince, and not a peer-reviewed research paper in the modern sense. Also, that the chi-squared test was not introduced into biology until after Mendel’s death.

## Mendel’s story

**Fig. 15:**
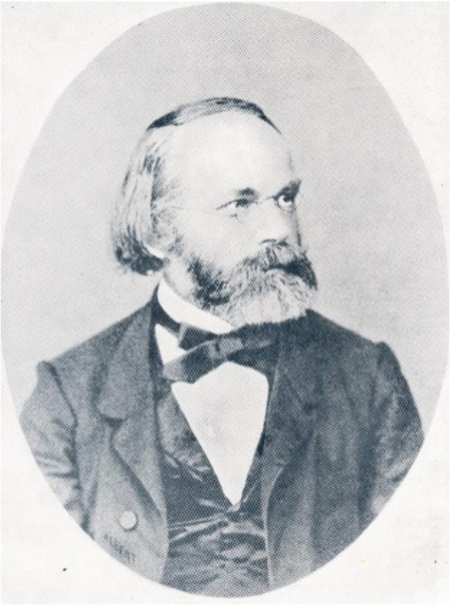
Mendel’s nemesis, Carl von Nägeli (1817–1891), his correspondent for 7 years. Who not only failed to repeat and to confirm Mendel on hand of seed samples and instructions provided by Mendel as it were on a “golden platter,” but then seems (conveniently?) to have forgotten that at one time he had had in hand a copy of Mendel’s paper with the letters and seed samples from Mendel (q.v. [5]). From Stubbe [21] with permission

When Mendel presented in 1865 and published the following year his was a totally unprecedented, “fantastic” story with sufficient strangeness so that no one in biology or botany over the next 34 years knew what to do with it. Or made the effort to understand or to follow Mendel, not even Nägeli (Fig. 15) with whom Mendel corresponded in detail over 7 years (1866–1873) and who, as professor of botany at the University of Munich, was more qualified than anyone else on the continent to do so. When Nägeli published his “idioplasma” theory in 1884, the year of Mendel’s death, he was met with less incomprehension than Mendel had been for a “story” far more fantastic than Mendel’s, seemingly having totally forgotten that he had once corresponded with Mendel, and had held the answer in his own hands [18].

The essence of Mendel’s story is of quintessential beauty and simplicity recalling von Baer [2]: *Simplex est sigillum veritatis*. In it Mendel:

- Anticipated the *genes*, or as he called them: *Die formbildenden Elemente*.
- Enuntiated clearly the concept later called *allelism*.
- Showed that the dominant and recessive (Mendel’s terms) members of a pair of alleles in a hybrid did *not blend* but remained discrete and intact and could *segregate* in future crosses.
- *Counted* the offspring of crosses between hybrids and found consistently a ratio of *3 dominants to 1 recessive* with 1/3 of the former purebreeding dominants and 2/3 hybrids, called heterozygotes nowadays, i.e. a ratio of 1:2:1 of AA to Aa to aa.
- Intuited that this 1:2:1 ratio in fact represented the expansion of the binomial (a + b)^2^ = a^2^ + 2ab + b^2^ one pair of alleles at a time.
- And that if this assumption were correct and each plant could be expected to yield a constant 4 seeds/generation, then continued selfing of heterozygotes (Aa, or 2ab combinatorially) should result in a gradual (asymptotic) diminution of the fraction of hybrids, i.e.

**Table:** 

	Generation			In a ratio of		
	"A"	"Aa"	"a"	AA	Aa	aa
1	1	2	1	1	:2	:1
2	6	4	6	3	:2	:3
3	28	8	28	7	:2	:7
4	120	16	120	15	:2	:15
5	496	32	496	31	:2	:31
n.				2^n^―1	:2	:2^n^―1

E.g. *n* = 10 : AA = 1023, Aa = 2 hybrids remaining, aa = 1023

**Fig. 16:**
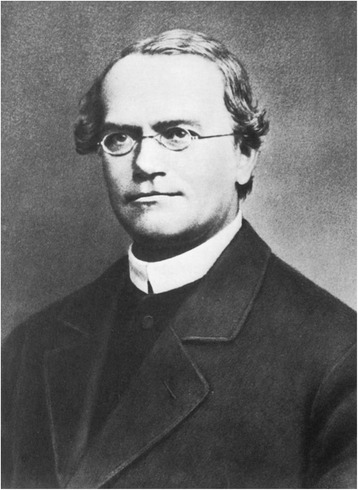
Mendel. From Stubbe [21], with permission

The fact that Mendel was able to express or paraphrase his results in clear and unambiguous mathematical terms makes them even more attractive and capable of proof of principle (statistical analysis). A^2^ + 2Aa + a^2^ was Mendel’s story behind the story. The great mathematician and computer pioneer (colleague of Babbage) Ada Lovelace (Byron’s daughter) said: “Mathematics [is] the language of the unseen relations between things, [hence] we must be able to fully appreciate, to feel, to seize, the unseen, the unconscious” in [11]. It was the genius of Mendel (Fig. 16) to have done so.

Thus, Mendel entered, quietly, that pantheon of the truly great in biology, those whose “stories” have become the foundations of that science relating to the development of individuals and of species. To name but a few:

- Aristotle (Harvey, Wolff): Epigenesis (the formed arises from the unformed);
- K.E. von Baer Pander, Wolff: Germ layers
- Agassiz: Glaciation
- Humboldt: Plant geography
- Wallace, Darwin: Natural selection
- Mendel: Segregation
- Mary L: Lyonization

**Fig. 17:**
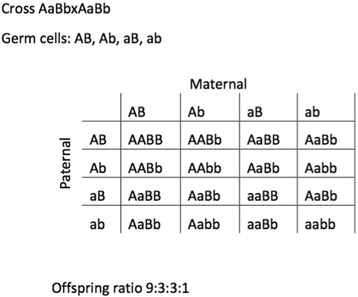
On the top: Punnett square of Mendelian dihybrid cross with resulting 9:3:3:1 ratio of offspring involving unlinked traits. Above: expected 9:3:3:1 ratio of offspring of a dihybrid cross AaBbxAaBb, represented in a Punnett square

**Fig. 18:**
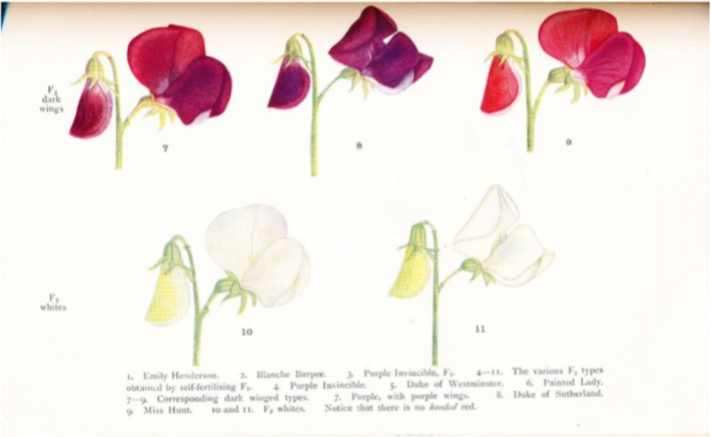
The 1905 results of Bateson and Punnett in sweet pea flowers (*Lathyrus odoratus*), a first hint of failure of independent assortment, thus of linkage, from [3], with permission

Finally, in his di- and tri-hybrid crosses, Mendel discovered that individual gene pairs in a more complex hybrid segregated *independently* of each other (given they were not linked) (Figs. 17 and 18).

Immediately after the rediscovery of Mendel in 1900 these data and their combinatorial consequences were confirmed and became the axiomatic cornerstone of genetics as genetics will become the cornerstone of medicine in the future going as we are already going from exome to genome sequencing. Mendel’s “shoes” were our baby shoes in genetics reminding us that if we don’t know where we came from we won’t know where we are going to.

Linkage. In 1905 Bateson and Punnett crossed 2 white types of sweet peas (“Emily Henderson”) which differed only in shape of pollen grain (normal long, and less common “roundish”). The initial focus was solely on the inheritance of pollen shape. But to their surprise, when they crossed the white hybrids between the 2 pollen types the offspring were purple-flowered “like the wild Sicilian plant from which our cultivated seed peas are descended” [3]. Even more remarkable was the fact that these 2 allelomorphs (white-purple blossoms; long-round pollen grains) did not reassort independently but appeared to show linkage, “coupling” as they called it, a phenomenon soon confirmed in *Drosophila*. This deviation from the 9:3:3:1 offspring ratio expected for a dihybrid cross of unlinked genes became the basis for later *mapping* studies, placing genes, in linear order, on the chromosomes, later aided by the discovery of the polytene (salivary gland) chromosomes in diptera.

## Colophon and conclusions

Orel [19, 20] in the epilogue to his retelling the story of Gregor Mendel, quoted Pope John Paul II commemorating Mendel’s death in 1984 to the effect that “many…have inclined more to admire the facts than to search for their reasons,” citing St. Augustine:

“The beauty of the earth is like a dumb voice arising from it. You observe it and look on its beauty, fertility and sources. You observe how the seed begins to germinate and gives a completely new thing which was sown. You take note of all of it, and it is as though you were asking in your mind why it is so. Filled with wonder, you search on, going to the root of things and finally you discover a great beauty and a magnificent strength.”

Ennaration in ps. 133, 13, 1876

Substitute Mendel for “you” in the above; Mendel, the Aesop of genetics.

Prof. John M. Opitz

## Footnotes

1 A-G: Mendel’s symbols, qv his 2^nd^ letter to Nägeli and his 1866 publication, capital vs lower case letters: convention introduced by Mendel to refer to dominant vs recessive character pairs (alleles) [5]

## References

[1] Ashliman DL (2003). Aesop’s fables.

[2] von Baer KE (1828). Über Entwickelungsgeschíchte der Thíere, Beobachtung und Reflexion. Part I.

[3] Bateson W (1909). Mendel’s principles of heredity. Cambridge at the University Press.

[4] Braess M (1920). Tierbuch.

[5] Correns C. 1905. Gregor /Mendels Briefe an Carl Nägeli. 1866-1873. Ein Nachtrag zu den veröffentlichten Bastardierungsversuchen Mendels. Abh mathem phys Klasse d Königl Sächsischen Ges d Wissensch. XXIX (3): 189-265. Leipzig bei B.G. Teubner. Correns was a student of Nägeli whose family made these 10 letters available to him. My copy of this reprint was inscribed by Correns to “J.G. Overton,” presumably before the First World War. Gift to John M. Opitz from the late Charles W. Cotterman of the University of Wisconsin. According to Rheinberger (2013) Correns married Elisabeth Widmer, Nägeli’s niece, in the year after Nägeli’s death. Overton is cited in Correns (1905) on the basis of his Über Parthenogenesis bei *Thalictrum purpurasceus* of 1904 in the Ber d Deutsch Bot Ges 22:274

[6] Darlington CD (1961). Darwin’s place in history.

[7] Darwin CR (1859). The origin of species by means of natural selection, or the preservation of favoured races in the struggle for life.

[8] Darwin CR (1868). The varieties of animals and plants under domestication (including the provisional hypothesis of pangenesis).

[9] Greenhill SD, Juczewski K, de Haan AM, Seaton G, Fox K, Hardingham NR (2015). Adult cortical plasticity depends on an early postnatal critical period. Science.

[10] Groll G (1953). Erzählungen aus Tausend und eine Nacht.

[11] Holmes R (2015). Enchantress of abstraction. Nature.

[12] Iltis H (1924). Gregor Johann Mendel, Leben, Werk and Wirkung.

[13] Margoliash D, Tschernichowski D (2015). Marmoset kids actually listen. Science.

[14] Mawer S. Gregor Mendel: Planting the seeds of genetics. New York, Abrams with The Field Museum, Chicago; 2006.

[15] Mendel G (1854). Brief an Dir. V. Kollar über Bruchus pisi. Verh zool-bot Verein, Wien (Sitz Ber).

[16] Mission Statement National Geographic Society. National Geogr Mag. 2015;288(4)

[17] Naudin C (1863). Nouvelles recherches sur l’hybridité dans les végétaux. Ann Sc Natur Bot.

[18] von Nägeli C (1884). Mechanisch-physiologische Theorie der Abstammungslehre.

[19] Orel V. Gregor Mendel, the first geneticist. Transl Finn S. Oxford: Oxford University Press; 1996.

[20] Orel V (1984). Mendel. Transl. Finn S.

[21] Stubbe H (1965). Kurze Geschichte der Genetik bis zur Wiederentdeckung der Vererbungsregeln Gregor Mendels.

[22] Takahashi DY, Fenley AR, Teramoto Y, Narayanan DZ, Borjon JI, Holmes P, Ghazanfar AA (2015). The developmental dynamics of marmoset vocal production. Science.

[23] Weiling F (1993). Johann Gregor Mendel, der Mensch und Forscher. Med Genetik.

